# Comprehensive Profiling and Inheritance Patterns of Metabolites in Foxtail Millet

**DOI:** 10.3389/fpls.2018.01716

**Published:** 2018-11-26

**Authors:** Shuangdong Li, Xuekui Dong, Guangyu Fan, Qiaofeng Yang, Jian Shi, Wei Wei, Fang Zhao, Ning Li, Xiaoming Wang, Feng Wang, Xiaolei Feng, Xiaolei Zhang, Guoliang Song, Gaolei Shi, Wenying Zhang, Fengcang Qiu, Dequan Wang, Xinru Li, Yali Zhang, Zhihai Zhao

**Affiliations:** ^1^Institute of Millet, Zhangjiakou Academy of Agricultural Science, Zhangjiakou, China; ^2^Wuhan Metware Biotechnology Co., Ltd., Wuhan, China

**Keywords:** *Setaria italica*, metabolic profiling, flavonoids, phenolamides, heterosis

## Abstract

Metabolomics aims at determining a sample's metabolites profile and hence provides a straight functional statement of an organism's physiological condition. Here, we investigated comprehensive profiling, natural variation and species-specific accumulation of both primary and secondary metabolites in foxtail millet using LC-MS, and inheritance patterns of metabolome in millet hybrids. The application of a broad target metabolomics method facilitated the simultaneous identification and quantification of more than 300 metabolites. The metabolic analysis of these compounds, such as flavonoids, phenolamides, hydrocinnamoyl derivatives, vitamins and LPCs, revealed their developmentally controlled accumulation, and natural variation in different tissues/varieties. Species-specific accumulation of secondary metabolites was observed based on a comparative metabolic analysis between millet and rice, such as flavonoid *O*-rutinosides/neohesperidosides and malonylated flavonoid *O*-glycosides. In analyzing the metabolic variations between hybrid progenies and their parental lines, including a photothermo-sensitive genic male sterility line and five Zhangzagu varieties, metabolic overdominant, and dominant patterns of inheritance could be observed. For example, hydrocinnamoyl derivatives and feruloylated flavonoids were identified as over-parent heterosis (overdominant) metabolites in milet hybrids. Our work paves the way for developing predictors of hybrid performance and the future analysis of the biosynthesis and regulation of relevant metabolic pathways in millet.

## Introduction

Foxtail millet (*Setaria italica*) is one of the oldest cultivated millet crops (Murugan and Nirmalakumari, 2006; Bidinger et al., 2007). Despite the fact that it is considered a minor cereal crop of regional importance, it is still widely cultivated as a dietary staple in the arid and semiarid regions in the world (Lata et al., 2013). Millets characteristically adapt to unfavorable ecological conditions, including untimely and irregular rainfall, high soil salinity and high temperature, and have remarkable nutritional properties (Doust et al., 2009). The nutritional quality of millet grains is usually equivalent or superior to that of other cereals containing high amounts of minerals, essential amino acids, carbohydrates, and vitamins (Lorenz and Hinze, 2002; Taira, 2002; Yang et al., 2013). However, its advantage has not been mirrored to the same extent in dissecting metabolic pathways because of the relative lack of knowledge of both primary and secondary metabolism in this species (Suma and Urooj, 2012).

Plant primary and secondary metabolites play vital roles in determining plant growth and development, pigmentation for fruits and flowers, plant interactions with microbes and animals, and plant defenses against abiotic stresses, etc. (Dixon and Paiva, 1995; Saito and Matsuda, 2010). For examples, as one of the most widespread groups of plant secondary metabolites, flavonoids have a multitude of biological functions, including protection against UV-light and phytopathogens, and male fertility (Caasi-Lit et al., 2007). Longer-term dietary administration of flavonols offers cardioprotection and improves the levels of cardiovascular disease risk factors in animals, and anthocyanins offer protection (Buer et al., 2010) against cancer, inhibiting the initiation and progression stages of tumor development (Wada and Ou, 2002). In plants, primary metabolites mainly include amino acids, nucleotides, fatty acids, carbohydrates and organic acids, while secondary metabolites include phenolic acids, flavonoids, polyamines, alkaloids, phytohormones and vitamins (D'Auria and Gershenzon, 2005; Schauer et al., 2008). To become established as a robust tool, metabolite profiling should be capable of covering a significant number of metabolites (Okazaki and Saito, 2012; Sumner et al., 2014). A broad target metabolomics method based on a new strategy stepwise MIM-EPI (stepwise multiple ion monitoring-enhanced product ions) has been developed recently using liquid chromatography-mass spectrometry (LC-MS) and has allowed an identification/annotation of more than 300 of them in rice (Chen et al., 2013). Metabolic profiling in millet, however, has only been reported in 4 months-old seedlings using gas-chromatography coupled with time-of-flight mass spectrometry (GC-TOF MS) to determine the diversity among 43 primary metabolites and five phenolic acids in three varieties (Jaekwang et al., 2013). The application of the stepwise MIM-EPI method in millet based on LC-MS therefore will allow for a comprehensive analysis of millet-specific metabolome.

Foxtail millet is a largely self-pollinating food crop and a forage species, while its low crossing fertility rates, and special floral morphology and anthesis behavior make foxtail millet one of the most difficult species to cross pollinate (Murugan and Nirmalakumari, 2006). The photothermo-sensitive genic male sterility line (PTGMS) represents a useful genetic tool for the development of two-line hybrids in foxtail millet (Yuan et al., 2008). Since the first PTGMS line 821 was developed in foxtail millet, many efforts have been made to develop commercial hybrid millet, including at least 14 Zhangzagu hybrid millets (Yuan et al., 2008). These hybrids have been grown widely in Northwest China and other tropical countries such as Africa due to their high yield production and adaptive capacities in response to marginal lands and various abiotic and biotic stresses. Heterosis describes the phenomenon of improved performance of a hybrid progeny compared to both homozygous parents and has been considered a central concept in plant breeding (Riedelsheimer et al., 2012). The potential role of metabolism has been largely overlooked in heterosis research despite its central role in growth and development. Significant differences have been found in the number and content of metabolites in cereals, such as rice and maize (Gong et al., 2013; Wen et al., 2014; Chen et al., 2016). A comprehensive metabolic analysis based on the millet progenies descended from crosses between PTGMS as the female parent and Zhangzagu varieties as the male parents will thus be helpful for developing predictors of hybrid performance and achieving the genetic breeding objectives in foxtail millet (Zhang et al., 2017).

In this study, comprehensive metabolic profiling and comparative analyses of both primary and secondary metabolites were performed in PTGMS and hybrid millets. A total of 314 out of 673 metabolites were tentatively identified and quantified using an LC-MS-based broad target metabolomics method. We were able to detect the developmental-specific accumulation and natural variation of metabolites, such as flavonoids, phenolamides, vitamins and LysoPCs. We were also able to detect the heterosis of metabolites when performing a metabolic analysis between the hybrid progenies and their parental lines. A comparative analysis of those metabolites between millet and rice suggests a species-specific accumulation of different metabolites.

## Materials and methods

### Plant materials

The six millet varieties used in this study were taken from a collection of cultivated Chinese germplasm, including photothermo-sensitive genic male sterility line A2 (PTGMS A2), Zhangzagu No. 3, No. 5, No. 6, and No. 13 and Dunhuang No. 2. Five millet progenies were descended from crosses between PTGMS A2 as the female parent and the five Zhangzagu varieties as male parents. The leaves at both the three-leaf and five-leaf stages were collected from those varieties and hybrid progenies. To investigate the photoperiod duration in relation to the controlled accumulation of metabolites at the five-leaf stage, the plants were grown in the dryland with an artificial photoperiod of 10 h/day for the short daylight condition and 14 h/day for the long daylight condition. The millet was grown in dryland in Zhangjiakou (Northern China). The rice was grown in the paddyfield in Wuhan (Southern China). Rice leaf samples were collected at the five-leaf stage from six varieties, including Nipponbare, Zhonghua 11, Minghui 63, Zhenshan 97, 9311 and Taishannuo. For each millet tissue sample, three samples were obtained for analysis by mixing four individual samples together. All the samples were harvested at 10:00–12:00 on that day, placed in liquid N_2_ immediately and stored at −70°C until vacuum freeze-drying.

### Chemicals

All the chemicals were of analytical reagent grade. Gradient-grade methanol, acetonitrile and acetic acid were purchased from Merck Company, Germany (http://www.merck-chemicals.com). The water was doubly deionised with a Milli-Q water purification system (Millipore, Bedford, MA). Authentic flavonoid standards were purchased from ANPEL, Shanghai, China (www.anpel.com.cn/cnw), BioBioPha Co., Ltd. (http://www.biobiopha.com/), and Sigma-Aldrich, USA (http://www.sigmaaldrich.com). Standards stock solutions were prepared using water, methanol and/or dimethyl sulfoxide (DMSO) as the solvent and stored at −20°C. Combined standard solutions of flavonoids were prepared just before use by mixing individual stock solutions and diluting these mixtures with 70% aqueous methanol.

### Sample preparation and extraction

The freeze-dried leaves were crushed using a mixer mill (MM 400, Retsch) with a zirconia beads for 1.5 min at 30 Hz. A 100 mg mass of powder was weighted and extracted overnight at 4°C with 1.0 ml of 70% aqueous methanol. Following centrifugation at 10,000 g for 10 min, the extracts were filtered (SCAA-104, 0.22 μm pore size; ANPEL, Shanghai, China, http://www.anpel.com.cn/) before LC-MS analysis.

### LC-MS conditions

The sample extracts were analyzed using an LC-ESI-MS/MS system (HPLC, Shim-pack UFLC SHIMADZU CBM30A system, www.shimadzu.com.cn/; MS, Applied Biosystems 4500 Q TRAP, www.appliedbiosystems.com.cn/). The analytical conditions were as follows, HPLC: column, Waters ACQUITY UPLC HSS T3 C18 (1.8 μm, 2.1 mm^*^100 mm); solvent system, water (0.04% acetic acid): acetonitrile (0.04% acetic acid); gradient program, 100:0 V/V at 0 min, 5:95 V/V at 10.0 min, 5:95 V/V at 11.0 min, 95:5 V/V at 11.1 min, 95:5 V/V at 15.0 min; flow rate, 0.35 ml/min; temperature, 40°C; and injection volume: 5 μl. The effluent was alternatively connected to an ESI-triple quadrupole-linear ion trap (Q TRAP)-MS.

LIT and triple quadrupole (QQQ) scans were acquired on a triple quadrupole-linear ion trap mass spectrometer (Q TRAP) using an API 4500 Q TRAP LC/MS/MS System, which was equipped with an ESI Turbo Ion-Spray interface operated in a positive ion mode and controlled by Analyst 1.6.2 software (AB Sciex). The ESI source operation parameters were as follows: ion source, turbo spray; source temperature 550°C; ion spray voltage (IS) 5,500 V; ion source gas I (GSI), gas II (GSII), curtain gas (CUR) were set at 55, 60, and 25.0 psi, respectively; and the collision gas (CAD) was high. Instrument tuning and mass calibration were performed with 10 and 100 μmol/L polypropylene glycol solutions in QQQ and LIT modes, respectively. The QQQ scans were acquired as MRM experiments with the collision gas (nitrogen) set to 5 psi. The DP and CE for individual MRM transitions were performed with further DP and CE optimization. A specific set of MRM transitions was monitored for each period according to the metabolites that were eluted within this period.

### Statistical analysis

Principal component analysis (PCA) was performed with log2 transformed metabolite data to improve the normality. For hierarchical clustering analysis (HCA) in the study of developmentally-controlled accumulation and natural variation of metabolites, metabolite data were firstly log2 transformed, followed by a min-max normalization. For HCA, the “heatmap.2” function in the “gplot” R-package was utilized to generate heatmap: Various R programming tools for plotting data, (http://cran.r-project.org/web/packages/gplots/index.html). Identification of differential accumulation of metabolites between different tissue/varieties were determined by partial least squares-discriminate analysis (PLS-DA) with the VIP values (Variable Importance for the Projection) >1, followed by both ANOVA (*p* ≤ 0.05) and fold-change (≥1.2 or ≤ 0.83). PCA and PLS-DA were performed with SIMCA-P version 14.0.

### Calculation of heterosis

Analyses of variance (ANOVA) were performed with the parental and hybrid generations. The absolute mid-parent heterosis (Hm) and Over-parent heterosis (Hp) were calculated as: Hm = (mean F1- mean MP)/mean MP where MP = (P1+P2)/2, Hp = (mean F1- max P) where max P refers to the higher performing parent, respectively (Barth et al., 2003).

## Results

### Metabolic characterization of zhangza hybrid millet leaves

A developed and broad target metabolomics method based on a strategy stepwise MIM-EPI was applied to the comprehensive profiling analysis of millet leaf metabolites using LC-ESI-MS/MS (Chen et al., 2013). To construct a millet leaf MS^2^ spectral tag (MS2T) library, leaf samples from six Zhangza hybrid millet varieties at both the three-leaf stage and the five-leaf stage were obtained and used for LC-MS analysis. For the data matrix generated by the library that contained more than 23000 signals with MS2 spectral in total, peaks were checked manually for signal/noise (s/n) > 10, and the redundant signals were removed as previously described. Finally, we obtained 673 highly reproducible metabolite signals with the product ion spectra (MS2). Based on the fragmentation pattern, the retention time (RT) and the mass-to-charge-ratio (m/z) values of each metabolite, 63 were putatively identified with that of the commercial standards, and 251 metabolites were annotated in cases when no authentic standards were available (Supplementary Tables S1, S2), which was performed as previously described (Chen et al., 2013; Dong et al., 2014). Among the results, apart from a few primary metabolites, we identified 36 amino acids with their derivatives, 19 nucleotide derivatives, 11 organic acids, and 20 glycerophospholipids. We also putatively identified a number of secondary metabolites, including 107 flavonoids, 18 hydrocinnamoyl derivatives, 17 phenolamides and 16 vitamin related compounds. A deep analysis of these flavonoids revealed that most of them were flavanone *C/O*-glycosides, flavone *C/O*-glycosides, flavonol *O*-glycosides and flavonolignans, coinciding with those that were previously reported in rice leaves (Dong et al., 2014). The annotation of eight flavone *O*-rutinosides/neohesperidosides and 10 malonylated flavonoid *O*-glycosides revealed a large accumulation of rutinosylated and malonylated flavonoids in millet.

### Developmentally-controlled metabolic accumulation in millet

**Figure 1:**
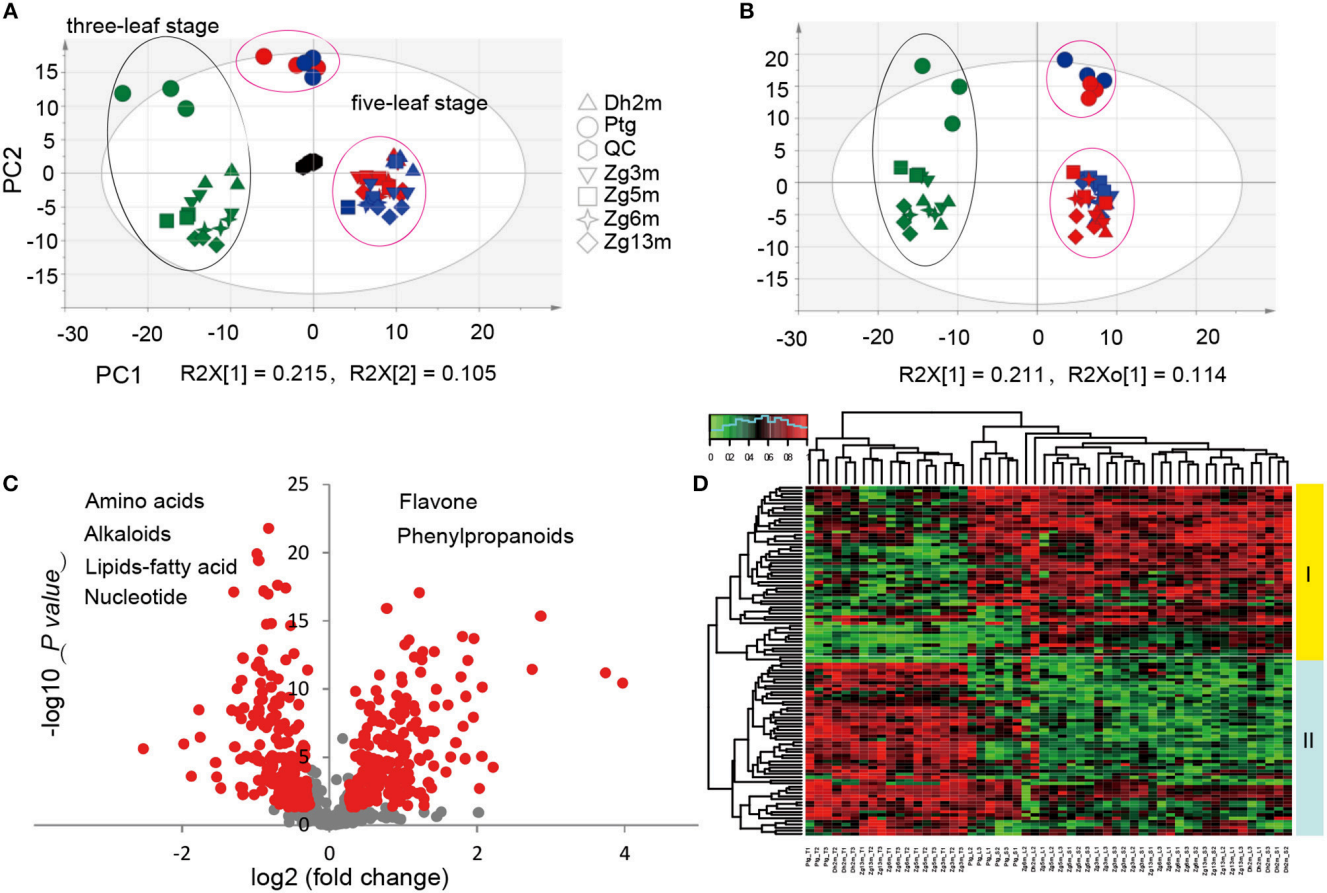
Statistical analysis of metabolic accumulation at different development all stages. **(A,B)** Score plot of PCA and PLS-DA for datasets from the three-leaf and five-leaf stage groups. The green color represents the sample at the three-leaf stage, the blue color represents the sample at the five-leaf stage under the short daylight condition, the red color represents the samples at the five-leaf stage under the long daylight condition, and the black color represents the quality control samples; **(C)** A volcano plot of the metabolic differences between the three-leaf and five-leaf stages (the red dot represents a metabolite with a fold change ≥1.2 or ≤ 0.83, *P-value* ≤ 0.05); and **(D)** a heat map of the relative differences in metabolites between the three-leaf stage and the five-leaf stage. The content value of each metabolite was normalized to complete the linkage hierarchical clustering. Red indicates high abundance, whereas the low relative abundance metabolites are green.

It has been suggested that plants produce dynamically changing metabolites in a developmental stage-dependent manner. To clarify the millet metabolic accumulation patterns during different developmental stages, the metabolic profile of leaf metabolites was compared for the samples that were collected from six millet varieties at both the three-leaf and five-leaf stages. Considering that the photoperiod duration might affect the accumulation of different metabolites, samples at the five-leaf stage were obtained under both short daylight and long daylight conditions. Based on the multiple reaction monitoring (MRM) mode using LC-ESI-QTrap4500, the high-throughput quantifications of a total of 673 metabolites were performed in the leaf samples from six millet varieties. In subjecting the metabolite data to principal component analysis (PCA), the first component (PC1, R2 = 0.215) separates the three-leaf stage (green color) and five-leaf stage samples (blue color, short daylight; red color, long daylight; Figure 1A), reflecting major differences in the metabolite levels between these two stages. However, a closer relationship between the samples under the short and long daylight conditions at the five-leaf stage indicated that there were smaller metabolic variations in the samples for different photoperiod duration conditions.

To identify the most meaningful changes in the metabolites, partial least squares-discriminate analysis (PLS-DA) was performed, and the VIP values (Variable Importance for the Projection, VIP≥1.0) were used (Figure 1B). The evaluation of the metabolite contents by ANOVA (*p* < 0.05) and fold-change revealed significant variations in 254 metabolites, of which 112 were identified/annotated. The visualization of their metabolic changes was performed by volcano plot as indicated by red dots, with the magnitude of the fold-change displayed along the X-axis and the statistical significance (–log10 of *p-value*) displayed on the Y-axis (Figure 1C). To explore their metabolic accumulation patterns, metabolites with levels that varied between the two stages were selected for hierarchical cluster analysis (HCA, Figure 1D). The accumulation of these metabolites displayed substantial variations in abundance within different tissues. Relative differences in the metabolites of different leaf samples grouped them into two primary clusters. A deep insight into those metabolites within cluster I revealed that most of them were flavonoid *C/O*-glycosides and flavonolignans, with higher levels at the five-leaf stage, while in cluster II, they were primarily represented by amino acids, nucleotide derivatives, nicotinic acid, pyridoxine and lipids, with higher levels at the three-leaf stage.

**Figure 2:**
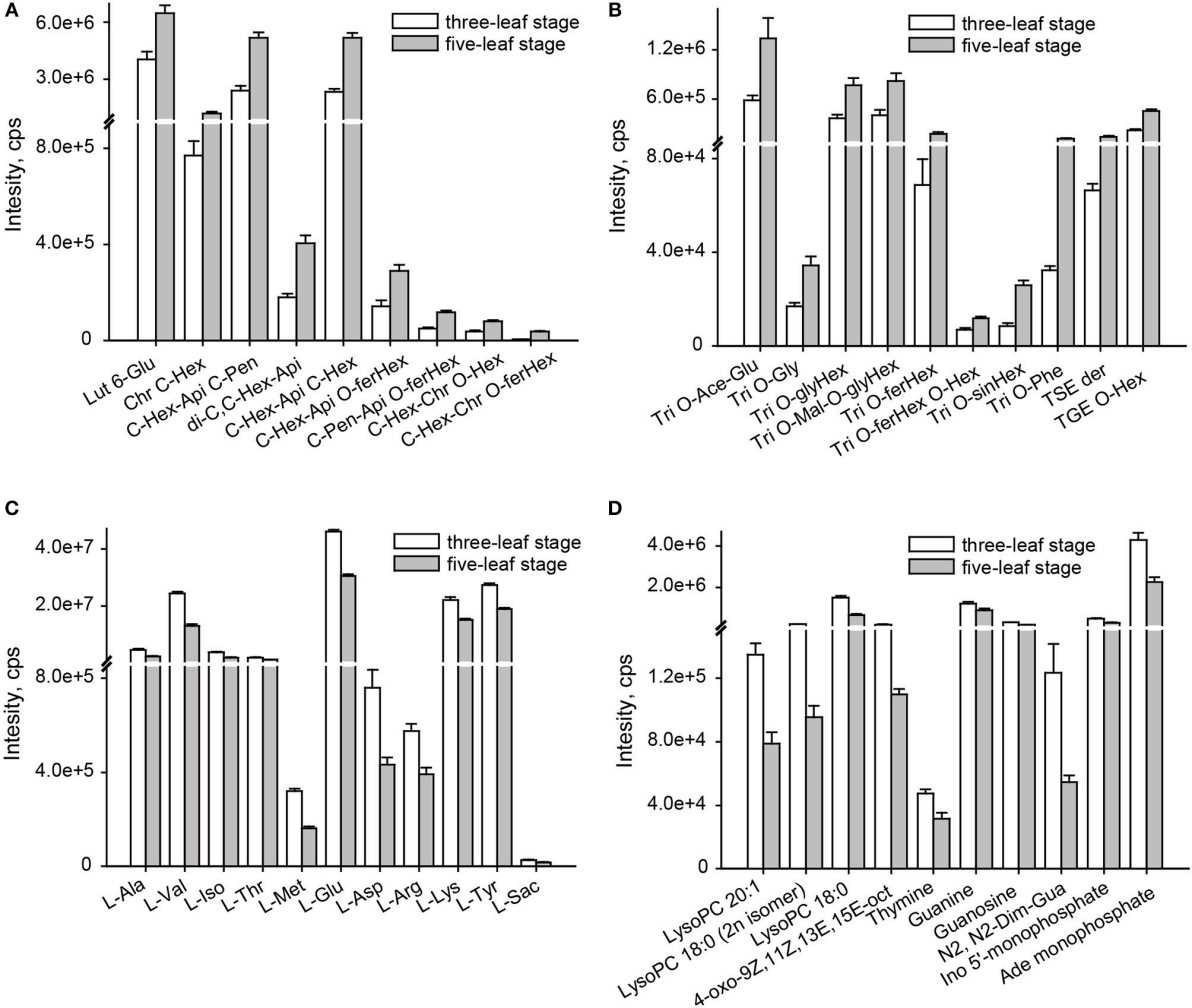
Change rate of metabolites altered at the three-leaf and five-leaf stages. **(A)** flavone C-aglycones; **(B)** flavone O-aglycones and flavonolignan; **(C)** amino acids; **(D)** lipids, nucleotide and its derivates. The identification of differential accumulation of metabolites between different tissue were determined by PLS-DA with the VIP values>1, followed by both ANOVA (*P* ≤ 0.05). Ace, acetyl; Ade, adenosine; Api, apigenin; Chr, chrysoeriol; Dim, dimethyl; Fer, feruloyl; GE, β-guaiacylglyceryl ether; Glu, glucoside; Gly, glycerin; Gua, guanosine; Hex, hexoside; Ino, Inosine; Lut, luteolin; mal, malonyl; oct, octadecatetraenoic acid; Phe, phenylformic acid; Pen, pentosyl; Sac, saccharopine; Sin, sinapoyl; Tricin, Tri; SE, syringyl alcohol ether.

A comparison of the flavonoid profiles indicated flavone *C-*glycosides, such as chrysoeriol *C-*hexoside, luteolin 6-*C-*glucoside and di-C,*C-*hexosyl-apigenin, and coumaroylated/feruloylated *C-*hexosyl flavone *O-*hexosides, such *C-*hexosyl-apigenin *O-*feruloylhexoside, *C-*hexosyl-chrysoeriol *O-*feruloylhexoside, *C-*hexosyl-luteolin *O-*feruloylhexoside and *C-*hexosyl-luteolin *O-*p-coumaroylhexoside, displayed the most significantly elevated levels at five-leaf stage (VIP≥1.0 and *p* ≤ 0.05), with elevated levels up to 2.0 and 3.1-fold, respectively (Figure 2A). Similar accumulation patterns were obtained for tricin derivatives, including acylated (acetyl, malonyl, glyceryl, phenylformoyl, and feruloyl) tricin *O*-glycosides and flavonolignans (Figure 2B). A total of 11 amino acids, namely L-alanine, L-valine, L-Isoleucine, L-threonine, L-methionine, L-glutamic acid, L-asparagine, L-arginine, L-lysine, L-tyrosine and L-saccharopine, and seven nucleotide derivatives, namely guanine, guanosine, thymine, N2, N2-dimethyguanosine, adenosine monophosphate, inosine 5′-monophosphate and beta-nicotinamide adenine dinucleotide, and four lipids (LPCs and fatty acids), LysoPC 18:0, LysoPC 18:0 (2n isomer), LysoPC 20:1 and 4-oxo-9Z,11Z,13E,15E-octadecatetraenoic acid, showed 0.6 times significantly decreased levels (Figures 2C,D). Both exhibited the vegetative stage dependent accumulation of both primary and secondary metabolites in millet leaves.

### Differential accumulation of metabolites among PTGMS A2 and zhangzagu lines

**Figure 3:**
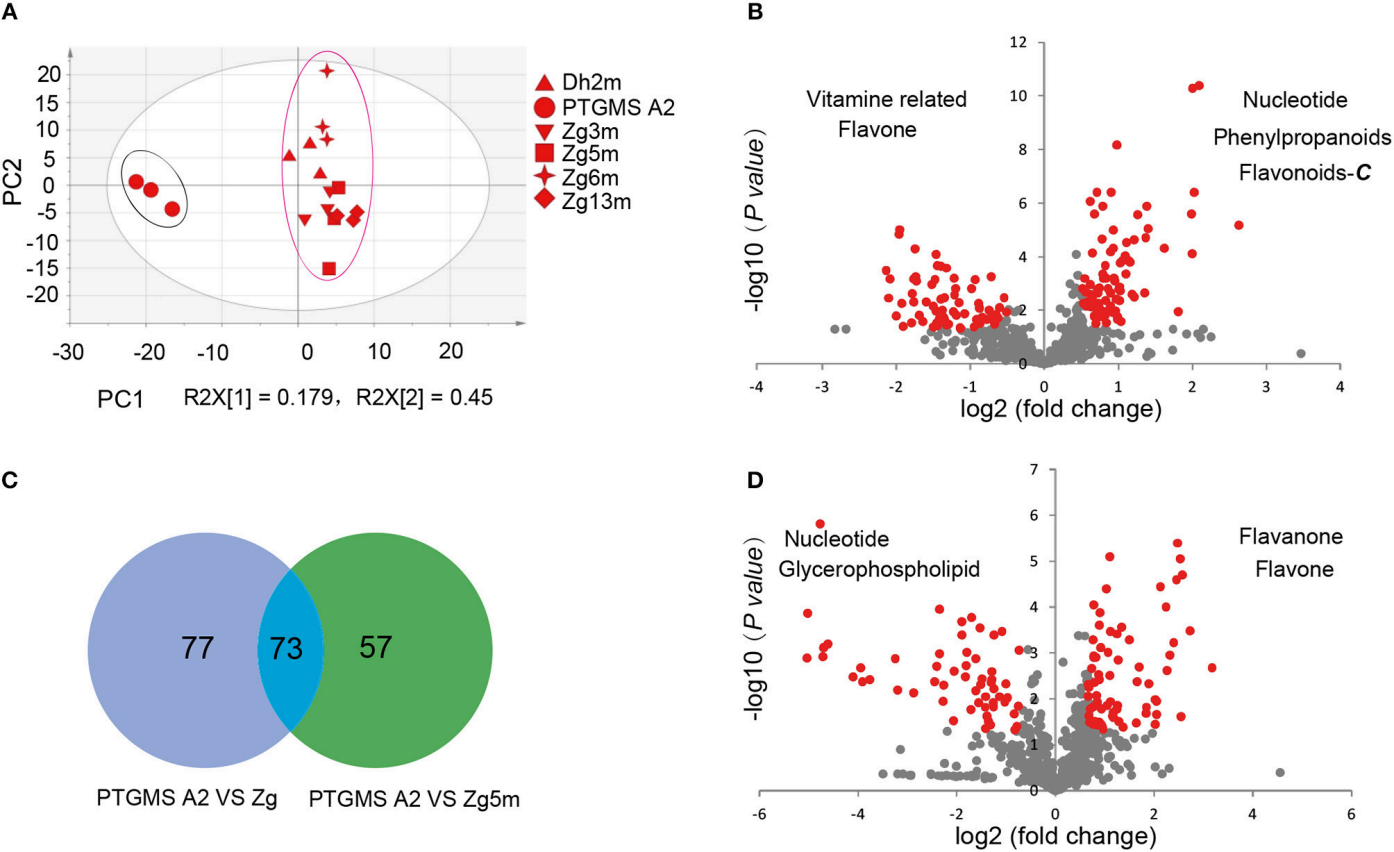
Differential accumulation of metabolites among the PTGMS A2 and Zhangzagu lines at the five-leaf stage. **(A)** The score plot of PCA for datasets at five-leaf stage under the long daylight condition. The different shape represents the different variety sample; and **(B,C)** a volcano plot of metabolic differences between PTGMS A2 and Zhangzagu **(B)**, and between PTGMS A2 and Zhangzagu No.5 **(C)** at the five-leaf stage. (the red dot represents a metabolite with a fold change ≥1.2 or ≤ 0.83, *P-value* ≤ 0.05). **(D)** Venn plot of the metabolic profiles of PTGMS A2 vs. Zg (group I) and PTGMS A2 vs. Zg5m (group II) at the five-leaf stage.

To investigate the metabolic accumulation patterns of millet varieties with different genetic backgrounds and geographical distributions, a comprehensive metabolic analysis of leaf samples from six millet varieties, namely Zhangzagu No. 3, No. 5, No. 6 and No. 13, and Dunhuang No. 2 and photothermo-sensitive genic male sterility line A2 (PTGMS A2), was performed. Among them, Zhangzagu No. 3, No. 6, and No. 13 and Dunhuang No. 2 are suitable for planting in dry land in the spring, whereas Zhangzagu No. 5 is suitable for use in irrigated land. PCA was visualized to uncover the range of the metabolic variations observed for those samples (Figure 1A). Similar patterns could be observed in the metabolic accumulation among these varieties between the three-leaf and five-leaf stages. Samples at the five-leaf stage were hence used, and a metabolic analysis was performed by PCA for the samples at long daylight condition (Figure 3A). The PC1 separated the PTGMS A2 and Zhangzagu No. 3, Zhangzagu No. 5, Zhangzagu No. 6, Zhangzagu No. 13 and Dunhuang No.2 clearly, while PC2 separated Zhangzagu No.5 and both PTGMS A2 and the other four Zhangzagu lines, indicting the natural variation in metabolites in the millet varieties.

A PLS-DA of metabolic accumulation between PTGMS A2 and the four Zhangzagu lines (group I), including Zhangzagu No. 3, Zhangzagu No. 6, Zhangzagu No. 13 and Dunhuang No. 2, which were studied as one group, was subsequently performed, as was the accumulation between the PTGMS A2 and Zhangzagu No. 5 lines (group II). The magnitude of the variation in these metabolites between the two groups was just as it was indicated in the volcano plot (Figures 3B,C). When comparing the metabolic accumulation between groups I and II, we observed a total of 73 metabolites that showed significant variations between PTGMS A2 and the Zhangzagu lines, while 77 and 57 metabolites showed significant variations between groups I and group II, respectively (Figure 3D).

Significantly higher levels of five nucleotide derivatives, that is adenosine, 5-methylcytidine, N2,N2-dimethyguanosine, 2'-Deoxycytidine-5′-diphosphate and beta-Nicotinamide adenine dinucleotide, and seven lipids, namely LysoPC 12:0, LysoPC 14:0, LysoPC 18:2, LysoPC 18:2 (2n isomer), LysoPC 18:3, 5,14,15-trihydroxy-6,8,10,12-eicosatetraenoic acid and linolenic acid methyl ester, could be detected in PTGMS A2 than that in the Zhangzagu varieties (Table 1). Similar patterns were obtained with respect to the accumulation of hydrocinnamoyl derivatives, including chlorogenic acid, 4-*O*-*p*-coumaroylquinic acid and coumarin; vitamin biosynthesis-related metabolites, including 4-pyridoxate and nicotinic acid; and phytohormones, including indole-3-acetic acid and *trans*-zeatin 7-glucoside. Six flavonoid *C-*aglycones, luteolin 6-*C-*glucoside, chrysoeriol *C-*hexoside, eriodictyol *C-*hexoside, hesperetin *C-*hexoside, *C-*hexosyl-chrysin *O-*feruloylhexoside, *C-*hexosyl-apigenin *O-*feruloylhexoside and *C-*hexosyl-luteolin *O-*p-coumaroylhexoside, also showed an over-accumulation in PTGMS A2, with elevated levels of up to 2.3-fold (Table 1). Moreover, the evaluation of flavonoid *O-*aglycones revealed a natural variation in their different modified derivatives in millet varieties. Among these derivatives, 10 flavonoid *O-*rutinosides/neohesperidosides displayed the highest accumulation levels in Zhangzagu No. 5, as in apigenin 7-*O-*rutinoside, apigenin 7-*O-*neohesperidoside, kaempferol 3-*O-*β-rutinoside and naringenin 7-*O-*neohesperidoside, while flavonoid *O-*malonylhexosides showed the lowest levels in Zhangzagu No.5, apigenin *O-*malonylhexoside, tricetin *O-*malonylhexoside and naringenin *O-*malonylhexoside (Table 1). However, vitamin B2 showed the highest levels in the four Zhangzagu varieties.

**Table 1:** Different metabolic accumulation among PTGMS A2, the Zhangzagu and zhangzagu No.5 lines.

**Compounds**	**PTGMS A2**		**The four Zhangzagu**		**Zhangzagu No.5**		**Fold change**	
	**Int. (cps)**	**SEM**	**Int. (cps)**	**SEM**	**Int. (cps)**	**SEM**	**PTG/ 4Zgs**	**PTG/ Zg5m**
**NUCLEOTIDE AND ITS DERIVATES**								
Adenosine	4.90E+04	3.30E+03	3.10E+04	2.40E+03	3.00E+04	3.40E+03	1.59	1.62
5-Methylcytidine	1.40E+04	2.60E+02	7.20E+03	5.10E+02	8.10E+03	1.40E+02	1.91	1.72
N2, N2-Dimethyguanosine	1.00E+05	1.20E+04	5.80E+04	5.40E+03	6.20E+04	4.90E+03	1.78	1.68
2′-Deoxycytidine-5′-diphosphate	2.10E+06	8.30E+04	1.30E+06	8.80E+04	1.10E+06	8.50E+04	1.54	1.83
Nicotinamide adenine dinucleotide	2.80E+05	3.60E+04	1.70E+05	1.50E+04	2.10E+05	3.30E+04	1.6	1.34
**LIPIDS**								
LysoPC 12:0	9.10E+03	9.50E+02	5.00E+03	4.20E+02	3.80E+03	4.20E+02	1.82	2.39
LysoPC 14:0	4.60E+05	1.60E+04	3.60E+05	2.40E+04	2.70E+05	3.40E+04	1.29	1.7
LysoPC 18:2	3.30E+07	1.80E+06	3.00E+07	1.40E+06	2.10E+07	2.20E+06	1.1	1.6
LysoPC 18:2 (2n isomer)	3.40E+07	1.70E+06	3.20E+07	1.20E+06	2.40E+07	2.20E+06	1.07	1.43
LysoPC 18:3	2.30E+06	1.80E+05	1.90E+06	1.40E+05	1.30E+06	9.10E+04	1.22	1.78
5,14,15-trihydroxy-6,8,10,12- eicosatetraenoic acid	9.00E+06	3.30E+05	8.80E+06	3.30E+05	6.50E+06	7.90E+05	1.02	1.38
γ-Linolenic Acid methyl ester	1.90E+05	5.70E+03	1.20E+05	1.20E+04	1.40E+05	3.70E+04	1.61	1.37
**HYDROCINNAMOYL DERIVATIVES**								
Chlorogenic acid	2.40E+05	2.60E+04	9.40E+04	8.50E+03	1.30E+05	1.50E+04	2.59	1.9
4-O-p-Coumaroylquinic acid	1.90E+06	2.90E+05	1.10E+06	1.10E+05	8.80E+05	9.60E+04	1.77	2.14
Coumarin	1.10E+07	3.00E+05	6.00E+06	4.90E+05	5.70E+06	1.30E+05	1.79	1.86
**VITAMINE RELATED**								
4-Pyridoxate	7.50E+05	2.80E+04	3.40E+05	3.50E+04	2.40E+05	2.70E+03	2.21	3.16
Nicotinic acid	3.60E+05	4.30E+04	1.90E+05	2.50E+04	1.60E+05	1.80E+04	1.9	2.26
**PHYTOHORMONES**								
Indole-3-acetic acid	4.80E+04	2.00E+03	3.20E+04	1.90E+03	3.40E+04	2.20E+03	1.49	1.41
*trans*-Zeatin 7-glucoside	4.60E+05	3.10E+04	2.80E+05	2.70E+04	3.80E+05	4.30E+04	1.66	1.2
**FLAVONOID** ***C*****-ALYCONES**								
Luteolin 6-C-glucoside	1.10E+07	1.40E+06	5.90E+06	6.50E+05	7.30E+06	4.90E+05	1.86	1.51
Chrysoeriol C-Hex	2.10E+06	1.40E+05	8.90E+05	7.70E+04	1.10E+06	1.40E+05	2.32	1.91
Eriodictyol C-Hex	5.30E+05	1.30E+04	2.90E+05	3.00E+04	3.40E+05	2.30E+04	1.87	1.58
Hesperetin C-Hex	1.50E+05	2.40E+03	1.00E+05	1.30E+04	9.40E+04	9.20E+03	1.44	1.59
C-Hex-apigenin O-ferHex	4.70E+05	3.50E+04	2.60E+05	3.80E+04	1.90E+05	6.00E+04	1.79	2.44
C-Hex-luteolin O-p-couHex	4.80E+04	9.00E+03	2.00E+04	2.70E+03	2.60E+04	2.10E+03	2.33	1.84
**FLAVONOIDS**								
Apigenin 7-O-neohesp	1.20E+06	8.80E+04	2.40E+06	5.00E+05	3.20E+07	9.20E+05	0.52	0.04
Apigenin 7-O-rutinoside	1.10E+06	8.00E+04	2.10E+06	4.50E+05	3.10E+07	2.90E+05	0.54	0.04
Chrysoeriol 7-O-rutinoside	4.50E+06	1.60E+05	3.30E+06	2.40E+05	4.30E+07	1.20E+06	1.37	0.11
Chrysoeriol O-Hex-O-Rha-O-Hex	2.00E+05	2.10E+04	1.70E+05	2.50E+04	4.00E+05	2.70E+04	1.22	0.51
Isorhamnetin O-rutinoside	5.40E+03	4.20E+02	8.00E+03	5.10E+02	8.10E+04	4.20E+03	0.67	0.07
Luteolin O-rutinoside	4.00E+05	4.10E+04	3.90E+05	2.90E+04	2.60E+07	7.80E+05	1.04	0.02
3′,4′,5′-Tricetin O-rutinoside	6.90E+03	4.60E+02	8.70E+03	7.40E+02	6.30E+04	3.90E+03	0.79	0.11
Tricin O-Rha-O-malHex	3.70E+05	1.10E+04	3.70E+05	1.70E+04	9.00E+06	1.80E+05	0.99	0.04
Tricin O-rutinoside	1.30E+06	1.90E+04	1.50E+06	5.50E+04	2.00E+07	7.00E+05	0.88	0.07
Tricin 4′-O-GE	3.00E+04	1.10E+04	4.30E+04	4.10E+03	1.70E+05	1.50E+04	0.71	0.18
Tricin 4′-O-GE O-rutinoside	1.20E+05	1.20E+04	1.10E+05	8.30E+03	2.00E+06	9.40E+04	1.05	0.06
Kaempferol 3-O-β -rutinoside	3.60E+05	2.50E+04	3.60E+05	2.50E+04	2.30E+07	2.40E+05	1.01	0.02
Quercetin 3-O-galactoside	9.50E+04	9.70E+02	6.20E+04	7.40E+03	9.70E+04	6.30E+03	1.54	0.98
Hesperidin	1.20E+05	9.10E+03	8.70E+04	4.90E+03	1.60E+06	7.80E+04	1.35	0.07
Naringenin 7-O-neohesp	3.40E+04	3.40E+03	5.50E+04	1.00E+04	8.80E+05	2.20E+04	0.62	0.04
Malvidin 3-O-glucoside	8.80E+03	9.10E+02	3.00E+04	2.90E+03	4.30E+04	4.90E+03	0.3	0.21
Chrysin O-Hex	6.40E+04	1.80E+03	5.50E+04	4.10E+03	2.70E+04	3.50E+03	1.16	2.42
Chrysin O-malHex	6.60E+05	8.10E+04	5.10E+05	2.90E+04	1.60E+05	8.10E+03	1.3	4.07
Apigenin O-malHex	1.90E+07	1.20E+06	2.60E+07	3.70E+06	4.50E+06	5.00E+04	0.7	4.14
Luteolin 7-O-glucoside	2.10E+07	2.30E+06	2.00E+07	1.40E+06	3.60E+06	2.50E+05	1.05	5.86
Luteolin O-malHex	1.10E+07	1.10E+06	7.60E+06	7.20E+05	1.20E+06	2.00E+05	1.46	9.05
Chrysoeriol O-Hex	4.70E+07	1.00E+06	3.50E+07	1.90E+06	8.60E+06	1.00E+06	1.35	5.48
Chrysoeriol O-malHex	5.30E+07	1.50E+06	3.90E+07	2.30E+06	8.90E+06	2.90E+05	1.37	5.95
Selgin O-Hex	1.60E+05	1.70E+04	1.80E+05	7.10E+03	8.80E+04	8.20E+03	0.87	1.79
Tricetin O-malHex	4.30E+06	1.30E+05	2.50E+06	1.30E+05	9.80E+05	4.40E+04	1.72	4.38
Eriodictyol 7-O-glucoside	7.90E+05	6.10E+04	6.70E+05	4.60E+04	2.40E+05	1.10E+04	1.18	3.25
Naringenin O-malHex	3.50E+06	3.10E+05	3.20E+06	4.10E+05	9.70E+05	4.50E+04	1.1	3.59
Vitamin B2	7.30E+05	1.50E+05	1.80E+06	9.60E+04	1.30E+06	5.70E+04	0.4	0.58

All data are given as mean ± SEM. fer, feruloyl; GE, β-guaiacylglyceryl ether; Hex, hexoside; mal, malonyl; neohesp, neohesperidoside; Rha, rhamnosyl;

### Overdominance and dominance pattern of metabolome revealed by PTGMS and zhangzagu hybrids

**Figure 4:**
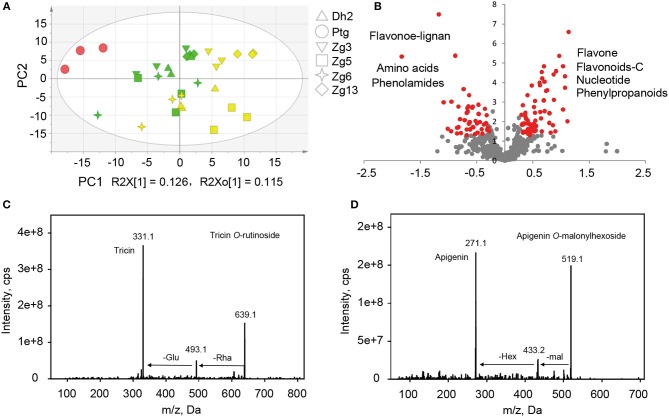
Metabolic profile analysis among hybrid progenies and the parental lines at the five-leaf stage. **(A)** The PCA score plot for datasets at the five-leaf stage. The green color represents the progenies, the red color represents the female parental line, and the yellow color represents the male parental lines, **(B)** A volcano plot of metabolic differences between the progenies and male parental lines at the five-leaf stage (the red dot represents a metabolite with a fold-change ≥1.2 or ≤ 0.83, *P*-value ≤ 0.05). **(C)** MS/MS spectra for tricin *O*-rutinoside; **(D)** MS/MS spectra for apigenin *O*-malonylhexoside. Glu, glucose; Hex, hexoside; mal, malonic acid; Rha, rhamnose.

To study the inheritance patterns of metabolites in millet, five hybrids were obtained by using PTGMS A2 as the female parent and the five Zhangzagu varieties (Zhangzagu No. 3, No. 5, No. 6, No. 13, and Dunhuang No. 2) as male parents. For both parental lines and the hybrid progenies, five leaf-stage samples (long daylight condition) were obtained and used for the metabolic analysis. The PCA showed that hybrid progeny samples lay between the female and male parents, revealing significant variations in the metabolites between the hybrid progenies and parental lines (Figure 4A). A shorter distance between the hybrid progenies and five male parents indicated that the progenies showed smaller metabolic variations for the Zhangzagu male parents than with the female parent. The separation of hybrid progenies between the four Zhangzagu varieties (Zhangzagu No. 3, No. 6, No. 13, and Dunhuang No. 2) and Zhangzagu No. 5 revealed by PC2 showed their significant differential metabolic accumulation, coinciding with significant metabolic variation among their parental lines.

PLS-DA was first performed to identify the metabolic variation between each hybrid progeny and parental line for Zhangzagu No. 3, No. 6, and No.13 and Dunhuang No.2 samples. The investigation of metabolites that showed similar accumulation patterns among the progeny and parental lines indicated that when the male parental lines were compared, the hydrocinnamoyl derivatives, including cinnamic acid, 4-*O*-*p*-coumaroylquinic acid, chlorogenic acid, feruloyl quinic acid and coumarin displayed 1.6 times significantly higher levels in the progenies (Supplementary Table S3). The accumulation of phenolamides with ferulated forms in the hybrid progenies, such as *N*-feruloyl agmatine, *N*-feruloyl spermidine and *N*′*,N*″-disinapoylspermidine, as well as feruloylated tricin, such as tricin *O*-feruloylhexoside and tricin *O*-feruloylhexoside *O*-hexoside, showed similar patterns. In particular, comparing the accumulation of a total of 11 flavonoid *O-*glycosides, such as tricin *O-*glucuronide, tricetin *O-*malonylhexoside, quercetin 3-*O-*galactoside, and syringetin *O-*hexoside, seven flavonoid *C-*glycosides and *C-*hexosyl flavone *O-*hexosides, such as luteolin 6-*C-*glucoside, chrysoeriol *C-*hexoside and *C-*hexosyl-apigenin *O-*hexoside, revealed their significantly elevated levels in the progenies in comparison to the male parent lines at the five-leaf stage, with elevated levels of up to 1.4-fold. However, coumaroylated putrescine, including *N*-*p*-coumaroyl putrescine and *N*-hexosyl-*p*-coumaroyl putrescine, showed significantly lower levels in the progenies than that in the male parent. Slightly decreased levels could also be detected for the tricin derivatives, such as tricin *O-*glycerin, and tricin 4′-*O-*(β-guaiacylglyceryl) ether 5-*O-*hexosyl-*O-*hexoside (Supplementary Table S3). A comparison between the progenies and female parent line was performed, and it showed that there were higher levels of feruloylated derivatives, such as ferulic acid O-hexoside, feruloyl quinic acid and the syringic acid O-feruloyl-O-Hexoside, in the progenies (Supplementary Table S3). Malvidin 3-*O*-glucoside, malvidin 3-*O*-galactoside, tricin *O*-glycerin, tricin 4′-*O*-(β-guaiacylglyceryl) ether and vitamin B2 showed similar results, while two vitamin-related compounds, including thiamin and 4-pyridoxate, phytohormone, including *trans*-zeatin 7-glucoside, and lipids, including LysoPC 12:0, LysoPC 16:2 and stearidonic acid, showed significant lower levels in the hybrid progenies compared to the female parent line (Supplementary Table S3).

To provide a further understanding of the genetic contribution to metabolic diversity, metabolic profile analyses of the over-parent heterosis (overdominant) and mid-parent heterosis (dominant) metabolites in hybrids were performed. The over-parent heterosis metabolites were determined for those metabolites that showed significantly variance in hybrid progenies than the parent lines, followed by heterosis of metabolites in the hybrids and parental lines. For the samples at the five-leaf stage, totals of 36 and 52 metabolites (18 and 26 identified/annotated) were determined for the over-parent heterosis and mid-parent heterosis metabolites, respectively. A deep insight into the over-parent heterosis metabolites at two stages showed that significantly higher levels of six hydrocinnamoyl derivatives and five feruloylated flavonoids could be detected in hybrid progenies, such as 4-*O-*p-coumaroylquinic acid, the syringic acid *O-*feruloyl-*O-*hexoside, N',N″-disinapoylspermidine, tricin *O-*feruloylhexoside, tricin *O-*feruloylhexoside *O-*hexoside and *C*-pentosyl-apigenin *O-*feruloylhexoside (Table 2). For the mid-parent heterosis metabolites, five hydrocinnamoyl acids and their derivatives, such as cinnamic acid and chlorogenic acid, and phenolamides, such as *N*-feruloyl agmatine and *N*-feruloyl spermidine could be identified. Moreover, mid-parent heterosis metabolites were also obtained for vitamin B2, indole-3-acetic acid, seven flavone/flavonol *O*-glycosides and three flavone *C*-glucosides (Table 2).

**Table 2:** Over-parent and mid-parent metabolites detected among the Zhangzagu hybrid lines.

**ID**	**Compounds**	**Male lines (M)**	**Female (F)**	**Progenies (P)**	**Average (F,M)**	**P/Average (F,M)**
			**Intensity (cps)**			**Ratio**
**OVERDOMINANT METABOLITES**						
mlm365	Ferulic acid O-Hex	3.5E+05	1.9E+05	4.4E+05	
mlm349	Feruloyl quinic acid	1.5E+06	1.0E+06	2.3E+06	
mlm318	4-O-p-Coumaroylquinic acid	1.1E+06	1.9E+06	2.0E+06	
mlm575	N-sinapoyl hydroxycoumarin	1.3E+05	1.1E+05	1.5E+05	
mlm275	Syringic acid-O-fer-O-Hex	9.8E+04	4.5E+04	1.2E+05	
mlm423	N', N″-disinapoylspermidine	1.5E+04	1.4E+04	2.0E+04	
mlm618	Tricin O-ferHex	3.3E+05	3.0E+05	5.7E+05	
mlm619	Tricin O-ferHex iso2	1.7E+05	1.6E+05	2.8E+05	
mlm603	Tricin O-ferHex O-Hex	1.3E+04	7.7E+03	2.2E+04	
mlm608	C-Hex-chrysin O-ferHex	3.1E+04	4.0E+04	4.3E+04	
mlm590	C-Pen-apigenin O-ferHex	1.0E+05	1.2E+05	1.4E+05	
mlm505	Chrysoeriol O-Hex- O-Rha-O-Hex	1.7E+05	2.0E+05	3.1E+05	
mlm589	Tricin 4′-O-GE O-Hex	3.5E+06	2.5E+06	3.8E+06	
mlm591	Chrysoeriol O-Hex- O-gluc-O-Hex	1.1E+05	1.3E+05	1.6E+05	
mlm493	Quercetin-3-O-glucoside	6.6E+04	9.2E+04	9.7E+04	
mlm484	Quercetin 3-O-galactoside	6.2E+04	9.5E+04	1.0E+05	
mlm015	Histidinol	4.9E+07	2.7E+07	4.9E+07	
mlm377	2-(anilinomethyl)pyrrolidine	1.2E+05	6.2E+04	1.4E+05	
**DOMINANT METABOLITES**						
mlm077	L-Methionine	1.6E+05	2.1E+05	2.0E+05	1.8E+05	1.07
mlm233	Xanthurenic acid O-Hex	1.6E+06	2.9E+06	2.5E+06	2.3E+06	1.11
mlm007	5-Methylcytosine	1.3E+04	7.5E+03	1.3E+04	1.0E+04	1.24
mlm627	Cinnamic acid	2.5E+04	3.7E+04	3.4E+04	3.1E+04	1.09
mlm329	Chlorogenic acid	9.4E+04	2.4E+05	1.8E+05	1.7E+05	1.09
mlm334	Coumarin	6.0E+06	1.1E+07	9.2E+06	8.3E+06	1.10
mlm305	N-feruloyl agmatine	3.2E+07	4.9E+07	4.6E+07	4.1E+07	1.13
mlm341	N-feruloyl spermidine	3.7E+06	7.5E+06	6.7E+06	5.6E+06	1.21
mlm607	Indole-3-acetic acid	3.2E+04	4.8E+04	4.0E+04	4.0E+04	1.00
mlm370	(-)-Riboflavin	2.0E+06	7.5E+05	1.7E+06	1.4E+06	1.23
mlm372	Vitamin B2	1.8E+06	7.3E+05	1.6E+06	1.3E+06	1.23
mlm629	Naringenin	1.2E+05	1.5E+05	1.5E+05	1.3E+05	1.10
mlm628	Apigenin	2.3E+05	8.5E+04	1.8E+05	1.6E+05	1.14
mlm399	Eriodictyol C-Hex	2.9E+05	5.3E+05	4.1E+05	4.1E+05	1.01
mlm486	Hesperetin C-Hex	1.0E+05	1.5E+05	1.4E+05	1.3E+05	1.10
mlm410	C-Hex-apigenin O-Hex	4.2E+06	6.3E+06	5.6E+06	5.2E+06	1.07
mlm631	Tricin	5.9E+06	3.5E+06	5.2E+06	4.7E+06	1.12
mlm367	Tricin di-O-Hex	1.2E+04	3.4E+03	9.4E+03	7.6E+03	1.24
mlm581	Tricin O-glucuronide	2.1E+05	5.5E+05	4.4E+05	3.8E+05	1.16
mlm622	Tricin O-glycerin	3.7E+04	9.5E+03	2.5E+04	2.3E+04	1.08
mlm633	Tricin 4′-O-GE	9.5E+06	4.8E+06	7.9E+06	7.2E+06	1.10
mlm327	Malvidin 3-O-glucoside	3.0E+04	8.8E+03	2.4E+04	1.9E+04	1.21
mlm323	Malvidin 3-O-galactoside	2.5E+04	1.2E+04	2.0E+04	1.9E+04	1.09
mlm565	Syringetin O-Hex	9.1E+04	1.4E+05	1.2E+05	1.1E+05	1.09
mlm208	Hydrastine	2.1E+04	4.8E+03	1.3E+04	1.3E+04	1.02
mlm094	Diaminopimelic acid	5.3E+05	2.1E+05	3.9E+05	3.7E+05	1.06

*Fer, feruloyl; GE, β-guaiacylglyceryl ether; gluc, glucuronic; Hex, hexoside; Pen, pentosyl; Rha, rhamnosyl*.

### Differential accumulation of metabolites within zhangzagu progeny

To investigate the metabolic accumulation between hybrid progenies and parental lines (Figure 4A), a further evaluation of the metabolite contents between the Zhangzagu No.5 hybrid progenies (Zg5p) and parental lines at the five-leaf stage by using PLS-DA and ANOVA was performed. A differential accumulation could be observed for the metabolites that showed significant variations between Zg5p and male parent lines when compared to the other four Zhangzagu lines (Figure 4B). The glycerophospholipids in the progenies, LysoPC 12:0, LysoPC 16:0, LysoPC 16:0 (2n isomer), LysoPC 18:2, LysoPC 18:2 (2n isomer), and LysoPC 18:3 showed 1.5 times higher levels than that in Zhangzagu No. 5 (Supplementary Table S4). Similar results were obtained for thiamin, *trans*-zeatin-*O-*glucoside, chrysoeriol *O-*hexosyl-*O-*glucuronic-*O-*hexoside, selgin *O-*hexoside, tricin *O-*rhamnoside and naringenin *O-*malonylhexoside. However, the accumulation of lower levels of tricin derivatives, including tricin *O-*phenylformic acid, tricin 4′-*O-*(syringyl alcohol)ether, and tricin 4′-*O-*(syringyl alcohol)ether derivative, 5-hydroxyindole-3-acetic acid and kynurenic acid in Zg5p than that in the male parent lines revealed their similar patterns in comparison to the other four Zhangzagu lines (Supplementary Table S4). When comparing them to the female parent line PTGMS A2, for those metabolites that displayed lower levels in Zg5p, they were primarily represented as three nucleotides, adenosine, beta-nicotinamide adenine dinucleotide and 2'-Deoxycytidine-5′-diphosphate; six flavonoid *O-*aglycones, namely naringenin *O-*malonylhexoside, hesperetin 5-*O-*glucoside, luteolin *O-*malonylhexoside, chrysoeriol *O-*hexoside, tricetin *O-*malonylhexoside, and tricin *O-*glucuronide; and three vitamin-related metabolites, 4-pyridoxate, 4-pyridoxic acid *O-*hexoside, and nicotinic acid-hexoside, partially coinciding with the result for the other four Zhangzagu lines (Supplementary Table S4).

The determination of over-parent heterosis and mid-parent heterosis metabolites in Zg5p and their parental lines revealed 24 and 15 metabolites (10 and 12 identified/annotated), respectively. The over-parent heterosis metabolites were primarily represented as ferulic acid *O-*hexoside; three flavonoids, tricin *O-*rhamnoside, tricin 4′-*O-*(β-guaiacylglyceryl)ether *O-*hexoside and quercetin 3,4′-*O-*di-beta-glucopyranoside; and two vitamin-biosynthesis related compounds, (S)-(+)-2-(anilinomethyl)pyrrolidine and thiamin; The mid-parent heterosis metabolites were represented as three flavonoids/anthocyanins, luteolin 6-*C*-glucoside, selgin *O*-hexoside and malvidin 3-*O*-glucoside; and five glycerophospholipids, that is LysoPC 16:0, LysoPC 16:0 (2n isomer), LysoPC 18:2, LysoPC 18:2 (2n isomer) and LysoPC 18:3, and *trans*-zeatin-*O*-glucoside (Supplementary Table S5), both suggesting a broad range of metabolic accumulation during hybrid selection.

### Species-specific metabolic accumulation between millet and rice

To obtain an overview of the metabolic variation within and between millet and rice, leaf samples from six millet varieties and six rice varieties were collected and used for broad target metabolic analysis. A comparison of the metabolic profiles between millet and rice revealed significant qualitative and quantitative variations in different metabolites (Table 3 and Supplementary Table S6). A total of six phenolamides, eight flavonoid *O*-rutinosides/neohesperidosides, such as tricin *O*-rutinoside [Figure 4C, m/z 331.1 is the diagnostic fragment ion of tricin, and the sequential nutral loss of 146 and 162 from 639.1 is the diagnostic fragment of rhamonose and glucose (rutinoside), respectively], and seven malonylated flavonoid *O*-glycosides, apigenin *O*-malonylhexoside (Figure 4D, m/z 271.1 is the diagnostic fragment ion of apigenin, The sequential nutral loss of 86 and 162 from 519.1 is the diagnostic fragment of malonic acid and hexose, respectively), displayed the most significantly higher levels in millet than that in rice, with elevated levels of up to 128.9, 61.0, and 27.9-fold, respectively (Table 3). Six hydrocinnamoyl derivatives, five vitamin related compounds and three phytohormones displayed 14.5, 8.2, and 2.6 times over-accumulation in millet, respectively (Table 3). The accumulation of primary metabolites, including a total of 16 LPCs and six nucleotide derivatives, showed similar patterns, with 3.9 and 9.0 times significantly higher levels in millet, respectively (Supplementary Table S6). When the levels of flavonoids with different moieties were compared, distinct species-specific accumulation patterns were observed. In contrast to the higher levels of flavonoid *O-*rutinosides/neohesperidosides and malonylated flavonoid *O-*glycosides in millet, flavone mono/di-*O-*glycoside along with their feruloylated derivatives, flavonolignan, flavone *C*-glycosides, *C*-glycosyl flavone *O-*glycosides, and malvidin 3-*O-*glycoside displayed significantly decreased levels relative to those in rice (Table 3 and Supplementary Table S6). Similar results were also obtained for *N*-*p*-coumaroyl spermidine, *N*-feruloyl putrescine, *N*-feruloyl cadaverine, and pyridoxine *O*-glucoside (Supplementary Table S6).

**Table 3:** Significant metabolic variation in leaves between millet and rice.

**ID**	**Compounds**	***P-value***	**Millet**	**Rice**	**Millet/Rice**
			**Intensity (cps)**		**Ratio**
**PHENOLAMIDES**					
mlm271	N-Coumaroyl agmatine	6.1E−04	4.7E+07	1.7E+05	285.1
mlm305	N-feruloyl agmatine	5.8E−06	5.2E+07	9.6E+06	5.4
mlm236	N-p-coumaroylputrescine iso1	2.5E−05	1.1E+07	3.7E+06	2.9
mlm252	N-p-coumaroylputrescine iso2	1.1E−09	1.4E+07	1.2E+06	11.7
mlm229	N-Hex-p-coumaroyl agmatine	2.1E−03	9.3E+05	1.8E+03	520.4
mlm244	N-Hex-feruloylagmatine	5.8E−03	6.0E+05	1.3E+04	47.3
**FLAVONOIDS**					
mlm583	Prunin	1.1E−05	1.3E+05	2.6E+04	5.1
mlm559	Naringenin 7-O-neohesperidoside	2.7E−04	2.2E+05	8.4E+03	26.5
mlm527	Apigenin 7-O-rutinoside	4.5E−03	4.2E+06	1.3E+04	334.4
mlm523	Apigenin O-rutinoside	2.3E−03	9.5E+06	2.1E+05	45.4
mlm552	Apigenin 7-O-neohesperidoside	1.4E−03	9.8E+06	4.0E+05	24.2
mlm449	Luteolin O-rutinoside	8.8E−04	5.4E+06	8.9E+04	60.2
mlm538	Chrysoeriol 7-O-rutinoside	2.6E−06	1.7E+07	3.0E+06	5.7
mlm536	Tricin O-rutinoside	1.9E−04	5.6E+06	3.4E+05	16.3
mlm594	Naringenin O-malonylHex	2.0E−03	3.3E+06	5.2E+04	64.2
mlm623	Chrysin O-malonylHex	2.2E−04	4.3E+05	2.2E+04	19.4
mlm596	Apigenin O-malonylHex	1.3E−04	2.5E+07	4.2E+05	60.3
mlm561	Luteolin O-malonylHex	1.1E−03	7.7E+06	6.4E+05	12.0
mlm600	Chrysoeriol O-malonylHex	2.8E−05	4.3E+07	3.4E+06	12.7
mlm597	Tricetin O-malonylHex	3.1E−06	3.1E+06	3.1E+05	9.8
mlm585	Tricin O-Rha-O-malonylHex	2.5E−03	2.1E+06	1.3E+05	16.9
**HYDROCINNAMOYL DERIVATIVES**					
mlm627	Cinnamic acid	1.5E−04	3.8E+04	1.2E+04	3.1
mlm320	coumaric acid	4.0E−02	2.9E+04	3.0E+03	9.7
mlm554	Ferulic acid	1.7E−03	1.4E+04	3.9E+03	3.4
mlm365	Ferulic acid O-Hex	1.5E−04	1.9E+05	2.4E+04	8.2
mlm225	Ferulic acid O-rutinoside	2.0E−03	3.1E+04	1.7E+03	18.2
mlm215	p-Coumaric acid-O-Rha-O-Hex	6.3E−03	4.6E+04	9.7E+02	47.8
mlm604	Coniferylaldehyde	1.1E−03	3.2E+05	5.1E+04	6.4
**VITAMINE RELATED COMPOUNDS**					
mlm243	D-Pantothenic acid	1.8E−06	1.1E+07	4.0E+06	2.7
mlm144	Nicotinic acid	3.6E−05	1.9E+05	5.0E+04	3.7
mlm206	4-Pyridoxate	6.6E−04	4.8E+05	2.3E+05	2.1
mlm460	Nicotinic acid methyl ester	2.6E−03	2.9E+04	8.6E+03	3.4
mlm377	(S)-(+)-2-(anilinomethyl)pyrrolidine	1.5E−02	1.0E+05	3.5E+03	28.9
**PHYTOHORMONES**					
mlm607	Indole-3-acetic acid	2.5E−02	3.5E+04	1.8E+04	2.0
mlm635	Methyl indole-3-acetate	9.8E−03	3.8E+03	1.2E+03	3.3
mlm258	*trans*-zeatin (tZ)	4.1E−04	2.0E+04	8.2E+03	2.4
**FLAVONOID O-ALGYCONES**					
mlm569	Luteolin 5-O-Hex	1.4E−05	1.5E+05	9.2E+05	0.16
mlm505	Chrysoeriol O-Hex-O-Rha-O-Hex	2.8E−04	1.4E+05	5.4E+06	0.03
mlm624	O-methylchrysoeriol O-Hex	2.6E−03	8.3E+03	1.5E+05	0.05
mlm565	Syringetin O-Hex	1.6E−02	6.8E+04	4.7E+06	0.01
mlm619	Tricin O-ferHex	1.1E−06	2.0E+05	1.1E+06	0.18
mlm612	Tricin O-sinapoylHex	2.6E−04	3.0E+04	9.9E+06	0.00
mlm367	Tricin di-O-Hex	2.2E−02	5.6E+03	1.1E+06	0.01
mlm603	Tricin O-ferHex O-Hex	1.4E−02	5.3E+03	9.4E+04	0.06
**FLAVONOLIGNAN**					
mlm571	Tricin 4′-O-GE	3.0E−02	1.8E+04	2.2E+07	0.00
mlm614	Tricin 4′-O-GE O-Hex	2.8E−03	1.1E+05	6.0E+06	0.02
mlm610	Tricin 4′-O-SE O-Hex	3.4E−03	4.9E+04	9.2E+05	0.05
**FLAVONOID C-AGLYCONES**					
mlm399	Eriodictyol C-Hex	5.3E−03	1.9E+04	2.3E+06	0.01
mlm393	C-Hex-apigenin C-Pen	2.0E−08	1.5E+06	3.6E+07	0.04
mlm590	C-Pen-apeignin O-ferHex	8.8E−03	2.8E+04	5.2E+06	0.01
mlm401	Luteolin 6-C-glucoside	8.5E−03	9.9E+05	5.4E+07	0.02
mlm346	C-Hex-luteolin O-Hex	1.0E−02	9.3E+04	2.5E+06	0.04
mlm455	C-Hex-luteolin O-p-couHex	1.9E−03	6.6E+03	2.2E+07	0.00
mlm462	C-Hex-luteolin O-ferHex	6.8E−03	1.5E+04	2.8E+06	0.01
**PHYTOHORMONES**					
mlm254	*trans*-zeatin-O-glucoside	1.4E−04	7.5E+04	2.3E+05	0.32
mlm357	*trans*-zeatin riboside (tZR)	1.5E−02	6.7E+03	1.6E+04	0.42
mlm309	*trans*-zeatin riboside-O-glucoside	6.1E−05	3.4E+04	8.7E+04	0.39

*Cou, coumaroyl; Fer, feruloyl; GE, β-guaiacylglyceryl ether; Hex, hexoside; Pen, pentosyl; Rha, rhamnosyl; SE, syringyl alcohol)ether*.

## Discussion

The combination of metabolite profiling with chemometrics has been widely used for food and crop products assessment and functional genomic research, further directing breeding strategies for improving and optimizing the balance of food components (Chen et al., 2016). The application of the broad target metabolomics method using LC-MS allows for the identification and quantification of several 100 metabolites within a single extract (Chen et al., 2014). Here we investigated the variations in the accumulation of over 300 primary and secondary metabolites in foxtail millet. Flavanones, flavones, flavonols, and anthocyanins could be detected within the flavonoids, and they mostly occurred as their glycosylated forms. Coinciding with previous reports in rice and wheat, flavone *C-*aglycones, including apigenin, luteolin and chrysoeriol, were identified as their mono *C-*hexoside, di-*C*,*C-*hexosides, *C-*hexosyl-*O-*hexoside and its feruloyl/coumaroyl derivatives (Brazier-Hicks et al., 2009). However, the flavone *O-*aglycones, showed a different accumulation between millet and other cereal crop, such as rice. In foxtail millet, the rutinosides/neohesperidosides of flavone/flavanone and malonylated flavonoid *O*-glycosides were found to be the major flavonoid *O*-aglycones constituents, and they showed significant higher levels than the levels in rice as described above. The further evaluation of flavonoid composition in foxtail millet revealed that flavonoid *O*-aglycones make up the major flavonoid constituents. More than 60 of them have been annotated. Investigations of the flavonoid compositions in rice have been reported, and they have shown that rice tends to accumulate higher levels of flavone *C*-aglycone, as well as flavonolignans (Schijlen et al., 2004; Dong et al., 2014).

Phenolamides are frequently referred as polyamines conjugated with hydroxycinnamic acid. Most of them occur as mono-, di-, or tri-phenolic acids (coumaric, caffeic, ferulic, and sinapoyl acid)-substituted polyamines, such as agmatine, putrescine, and spermidine (Bassard et al., 2010; Quinet et al., 2010). Phenolamides have long been shown to play important roles in a wide range of biological processes, including plant development, pathogen resistance, and defense against abiotic stresses, such as mineral deficiencies, dehydration, salt stress, and more recently UV irradiation (Bassard et al., 2010). The over-accumulation of phenolamides could be detected in foxtail millet when compared to that in rice, probably due to their arid growing conditions. Glycosylated conjugates of phenolamides, such as *N*-hexosyl-feruloyl agmatine and *N*-hexosyl-*p*-coumaroyl agmatine, could be found in millet at up to 100-times higher levels than that in rice. Under most abiotic stress conditions, cellular polyamine and phenolamide levels were changed (Wang et al., 2015). The investigation of the polyamine profile in 21 rice cultivars under moderate long-term drought stress demonstrated a coordinated adjustment for the accumulation of spermine under drought conditions (Do et al., 2013). Under salt stress, the free polyamine cellular levels increased in the salt-resistant rice cultivar, as did their biosynthetic enzymes, such as arginine decarboxylase and ornithine decarboxylase (Quinet et al., 2010).

The tissue-specific and developmentally-controlled accumulation of both flavonoids and phenolamides in rice has recently been studied. Leaf tissues contained the highest levels of most flavonoids and phenolamides (Dong et al., 2014, 2015). An evaluation of the contents of these metabolites indicated that there were significantly elevated levels in the leaves at the early vegetative stage. Similar patterns could be observed for the accumulation of flavonoid *C*/*O*-glycosides, flavonolignans and three phenolamides in foxtail millet based on our study. By contrast, the accumulation of amino acids and nucleotides showed decreased levels. Being essential nutrient for human consumption, amino acids are also the substrates for the biosynthesis of a wide variety of secondary metabolites in plants, such as phenylalanine and arginine, which are known as the starting points of flavonoids and polyamines, respectively (Taira, 2002). Therefore, an increase in these secondary metabolites is probably due to the consumption of primary metabolites. Being sessile in nature, plants are forced to thrive under stressful conditions (Lata et al., 2010). An increase in secondary metabolites, such as flavonoids and phenolamides, at the early vegetative stage might be crucial for environmental acclimatization and plant survival (Aidoo et al., 2016; Zhang et al., 2017).

In addition to the various tissues, the differential accumulation of metabolites in millet was also found between the PTGMS and Zhangzagu lines, indicating natural variation of primary and secondary metabolism in millet. The over-accumulation of flavone *O*-hexosides and some phenolamides in the Zhangzagu varieties implied that they had important roles in drought tolerance, since most of the Zhangzagu varieties are suitable to be planted in dry land. For flavonoid *O*-aglycones that showed significantly elevated levels in the PTGMS line, they were primarily represented as malonylated derivatives. Similar patterns could be detected in the accumulation of two phytohormones, including methyl indole-3-acetate and *trans*-zeatin 7-glucoside, and vitamin-related compounds, both suggesting that they were probably involved in photoperiod response and plant fertility. This suggestion is evidenced by a further comparison of the metabolic variation between the parental lines and their hybrid progenies. We found that indole-3-acetic acid, *trans*-zeatin-*O*-glucoside, some vitamins and LysoPCs were identified as mid-parent heterosis metabolites. The natural variation of both primary and secondary metabolites was observed in different foxtail millet varieties and their hybrid progenies based on our study. The rice metabolome reportedly showed significant natural variation in its core germplasms (Chen et al., 2013). Secondary metabolites, such as *C*-glycosylated flavones, malonylated flavonoid *O*-aglycones and phenolamides with different modification moieties, showed significant variations between the *japonica* and *indica* rice subspecies, and they represented good candidate metabolic markers for distinguishing the rice cultivar identity within a diverse collection of rice accessions (Dong et al., 2014; Johnson et al., 2016). One of the last steps of these is the formation of the glycosidic bond between flavone/flavonol and sugar moieties for flavonoid *O*-glycoside, or the formation of the ester bond flavonoid *O*-glycoside and hydroxycinnamoyl-CoA catalyzed by members of the BAHD acyltransferases for malonylated flavonoids. The most significant higher accumulation of secondary metabolites, such as flavonoid *O*-rutinosides/neohesperidosides and malonylated flavonoid *O*-glycosides, in millet than that in rice could possibly be the results of enriched gene duplication event in foxtail millet (Chen et al., 2016; Peng et al., 2017). A protein phylogeny of the candidate flavonoid glycoylatransferase and the BAHD acyltransferase in millet, rice and maize, etc., could be constructed for further illustrating the possible genetic basis for controlling the accumulation of these metabolites.

Metabolic profiling analysis has been used for evaluation of inheritance patterns in inbred lines or segregating populations, and prediction of complex heterotic traits in plants (Schauer et al., 2006; Riedelsheimer et al., 2012). To provide information on the genetic control of the millet metabolome, metabolic analysis of parental lines and hybrid progenies was performed. Analyzing heterosis pattern of the different metabolites in millet hybrids, both positive dominance and overdominance patterns were observed. A deep look into metabolites with positive overdominance patterns indicated that they were primarily represented by feruloylated flavonoid/phenolamide derivatives. The positive heterotic effects of these metabolites seem conserved throughout all crosses, which implied that hybridization might favor a broad range of flavonoid, phenolamide and phenolic acid accumulations in millet. Cereal polyphenols have always received a great deal of attention due to their important roles in resisting to biotic and abiotic stresses (Jun et al., 2012). These compounds are present in free and conjugated forms, such as sugars, flavonoids and phenolamides, which alter their solubility and thus their bioavailability and bioactivity. This is probably supported by the predominantly positive heterosis accumulation for most amines and amino acids observed in the root of maize hybrids (Lisec et al., 2011). However, the comparative analysis of the root metabolome of six parental maize inbred lines and their 14 corresponding hybrids illustrated complex metabolite inheritance patterns. For most metabolites, they followed an overdominance inheritance pattern the majority displaying a negative overdominance, such as sugars and members of the central energy metabolism (Lisec et al., 2011). It has been reported in Arabidopsis plant most of the central energy metabolism related metabolites display a negative correlation with biomass due to draining the pools for these metabolites to promote growth (Meyer et al., 2007). In contrast, based on 76 introgression lines, assessment of inheritance mode of metabolic quantitative trait loci (mQTL) in tomato indicated that this is the not case. Vast majority of the mQTLs of secondary metabolism exhibits dominant modes of inheritance with only a minority displaying recessive modes of inheritance and no incidence of overdominance (Alseekh et al., 2015). Despite of these, heritability analyses on the basis of the individual compounds revealed different features. Two acyl-sugars, 10 flavonoids and 11 hydroxycinnamates displayed high heritability in tomato fruit prericarp. Further heritability analyses revealed that mQTLs of secondary metabolism were less affected by environment than mQTLs of primary metabolism. This probably explains why overdominant metabolites found in millet were mostly represented as secondary metabolites based on our study, both directing metabolic engineering of the levels of these metabolites via breeding.

An examination of the qualitative and quantitative differences in both the primary and secondary metabolites present in different tissues and varieties of millet has allowed us to study their comprehensive profiles, and work toward a better understanding of their functions(de Oliveira Dal'Molin et al., 2016). Core collections millet resources have been widely used to determine their population structure and to investigate important agronomic and abiotic stress-related traits (Wang et al., 2012). With progress in genome sequencing and high-throughput genotyping, genome-wide association studies have been performed to investigate the genetic control of natural variation in agronomic traits in foxtail millet (Zhang et al., 2012; Jia et al., 2013). Metabolites are regarded as a bridge between the genome and the phenome (Chen et al., 2016; Zhang et al., 2017). An integration of metabolomics strategies with GWAS in millet will provide fundamental resources for functional genomics research and genetic improvement (Saito, 2013; Chen et al., 2014; Luo, 2015).

## Author contributions

ZZ designed the research. SL and GF supervised this study. XZ, GuS, GaS, WZ, FQ, DW, XL, and YZ. participated in the material preparation. JS, WW, FZ, XW, FW, and XF carried out the metabolite analyses. XD, NL, SL, QY, and GF performed the annotation of the metabolites and performed the data analysis. XD and SL discussed the results and wrote the manuscript.

### Conflict of interest statement

XD, QY, JS, and NL were employed by company Wuhan Metware Biotechnology Co. The remaining authors declare that the research was conducted in the absence of any commercial or financial relationships that could be construed as a potential conflict of interest. The reviewer CW and handling Editor declared their shared affiliation.
